# Molecular Advances and Sustainable Strategies in Mushroom Production for Food Security: A Review

**DOI:** 10.3390/jof12030205

**Published:** 2026-03-11

**Authors:** Dali V. Francis, Malu Kishorkumar, Zienab F. R. Ahmed, Elke G. Neumann, Shyam S. Kurup

**Affiliations:** Department of Integrative Agriculture, College of Agriculture and Veterinary Medicine, United Arab Emirates University, Al Ain P.O. Box 15551, United Arab Emirates

**Keywords:** mushrooms, sustainability, arid land, gene editing, circular economy, lignin, food security

## Abstract

Mushrooms offer a promising solution for sustainable food production due to their nutritional value, low resource requirements, and ability to grow in diverse environments. As interest in mushrooms grows, it is important to understand where current research is focused and where key gaps remain. A bibliometric analysis of 776 research articles indexed in Web of Science revealed a strong emphasis on yield, substrate reuse, and enzymatic degradation, but limited attention to molecular approaches, climate adaptation, and studies from arid regions such as the Middle East. Building on these findings, this review explores the ecological diversity of mushrooms and their adaptations across tropical, temperate, boreal, and arid ecosystems. It discusses the role of mycorrhizal and microbial interactions in nutrient cycling and environmental resilience, including desert truffle symbioses. Key pathways and genetic regulation involved in lignin degradation are outlined, along with recent advancements in transcriptomics, proteomics, genomics, metabolomics, and metagenomics that support improved cultivation and bioactive compound production. The review also addresses sustainable practices, such as microbiome integration and resource recycling, to enhance mushroom farming. The aim is to bring together ecological insights and molecular strategies to support sustainable mushroom production, particularly in regions facing resource and climate challenges.

## 1. Introduction

Mushrooms represent a unique blend of ecological sustainability and nutritional value, positioning them as a valuable contributor to food system diversification and resilience, particularly in resource-constrained settings. As heterotrophic organisms, mushrooms thrive on agricultural residues, converting lignocellulosic biomass into high-protein food and bioactive compounds [1]. This conversion is facilitated by extracellular enzymatic systems, including laccases, manganese peroxidases, lignin peroxidases, cellulases, and hemicellulases, which enable the depolymerization of lignin, cellulose, and hemicellulose into assimilable nutrients. This ability to utilize waste not only reduces the environmental burden but also adds economic value, especially in regions with limited arable land and water resources. Consequently, mushrooms have emerged as a critical component in sustainable agricultural systems, capable of addressing critical challenges in both food and environmental security [2].

The nutritional profile of mushrooms, rich in proteins, vitamins, minerals, and antioxidants, underscores their potential as a sustainable food source [3]. On a dry-weight basis, edible mushrooms typically contain approximately 15–35% protein, 20–40% dietary fiber, and low lipid content (generally <5%), while also serving as important sources of potassium, phosphorus, iron, zinc, and B-group vitamins [3]. Economically, mushroom cultivation offers low-input, high-output agricultural models, particularly suitable for smallholder farmers. Species like *Pleurotus ostreatus* [4] and *Agaricus bisporus* [5] are widely cultivated due to their adaptability to diverse substrates and climates, making them ideal for resource-constrained regions. Moreover, mushrooms contribute significantly to ecosystem services through their role as natural decomposers, breaking down complex organic polymers such as lignin and cellulose to enable nutrient recycling in soils. This decomposition capacity is especially pronounced in white-rot and litter-decomposing fungi, which possess oxidative and hydrolytic enzymes responsible for efficient lignin mineralization and cellulose degradation. This lignocellulose degradation capability supports their growth and has implications for bioremediation and sustainable waste management. Spent mushroom substrate (SMS), a by-product of cultivation, can be repurposed as a soil conditioner or bioenergy source [6].

Despite their ecological and economic importance, research on mushrooms has traditionally been fragmented across disciplines. To better understand these trends and identify gaps, this review employs a detailed bibliometric analysis to evaluate the global research landscape. The findings of this analysis provide a foundation for exploring molecular advancements, such as transcriptomics, proteomics, and synthetic biology, and their integration with sustainable practices like circular economy models. By bridging these perspectives, the review aims to explore the genetic and enzymatic mechanisms underlying mushroom growth, propose innovative strategies for improving cultivation systems, and highlight emerging trends and research gaps in the field. Ultimately, this work aims to present mushrooms as an effective solution to the combined challenges of food security and environmental sustainability.

## 2. Bibliometric Analysis

**Figure 1:**
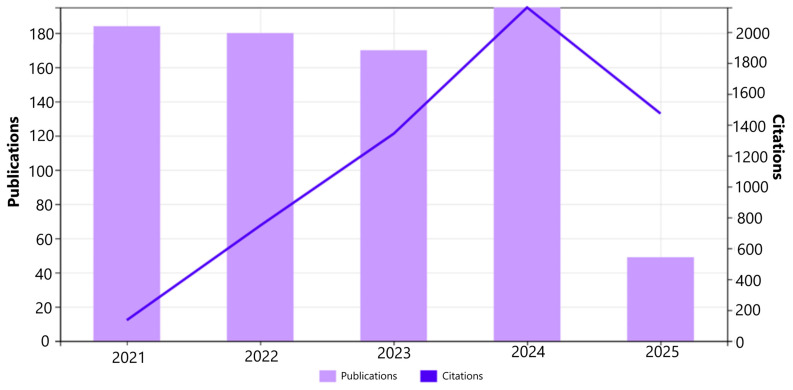
Annual publications and citations on mushroom research from 2021 to 2025 (Source: Web of Science Core Collection analytics).

A bibliometric analysis was carried out to examine recent research trends in mushroom cultivation, sustainability, and emerging molecular approaches. Data were retrieved from the Web of Science Core Collection using the search string *“mushroom AND (cultivation OR production) AND (sustainab OR molecular OR omics OR biotechnology)”**, applied to the title, abstract, and author keyword fields. Although broader taxonomic terms such as fungi, Basidiomycota, and Ascomycota were not explicitly included, the search strategy was designed to capture cultivation- and production-focused studies where mushrooms represent the primary applied system. Only peer-reviewed research articles published in English between 2021 and 2025 were included, yielding 776 records. The five-year time window was selected to reflect recent growth in molecular, omics-based, and sustainability-focused mushroom research, rather than to provide exhaustive historical coverage. Records were exported and analyzed using bibliometrix R package Biblioshiny interface run in RStudio version 2023.12.1 and VOSviewer version 1.6.20. During data preparation, duplicates were checked and removed where present, and author keywords were lightly cleaned by merging obvious spelling variants and synonyms prior to keyword mapping. Titles and abstracts were screened, and studies not relevant to cultivation/production, sustainability, or molecular/omics approaches were excluded. The final dataset was used to evaluate publication trends, keyword relationships, thematic structures, and country-wise research output [7,8]. As shown in Figure 1, the number of publications increased steadily from 2021 to 2024, with a peak in 2024. The lower publication count observed for 2025 reflects incomplete indexing at the time of data retrieval rather than a confirmed reduction in research activity. Citation trends followed a similar upward pattern, indicating sustained and growing interest in mushroom-related research [9].

**Figure 2:**
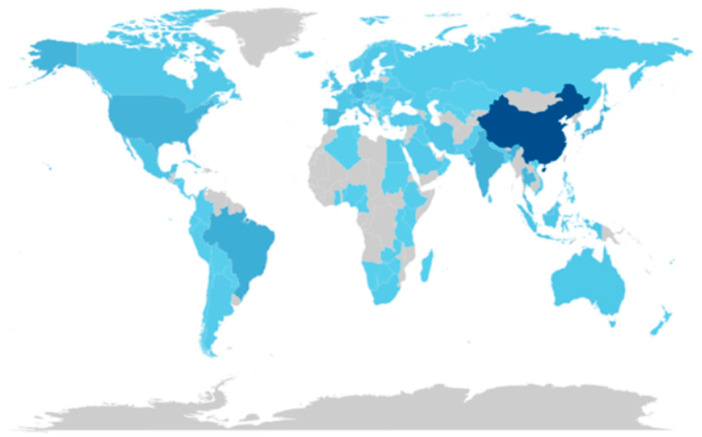
Country-wise scientific production in mushroom research (2021–2025) (RStudio bibliometric tools). Darker blue indicates higher publication output; lighter blue indicates lower output.

Country-level analysis (Figure 2) shows that research output is dominated by China, India, Brazil, and the United States. In contrast, contributions from the Middle East, including the UAE, remain limited. This uneven geographical distribution highlights a clear gap in research focused on arid and semi-arid regions, supporting the need for studies addressing mushroom production under dryland conditions [10].

**Figure 3:**
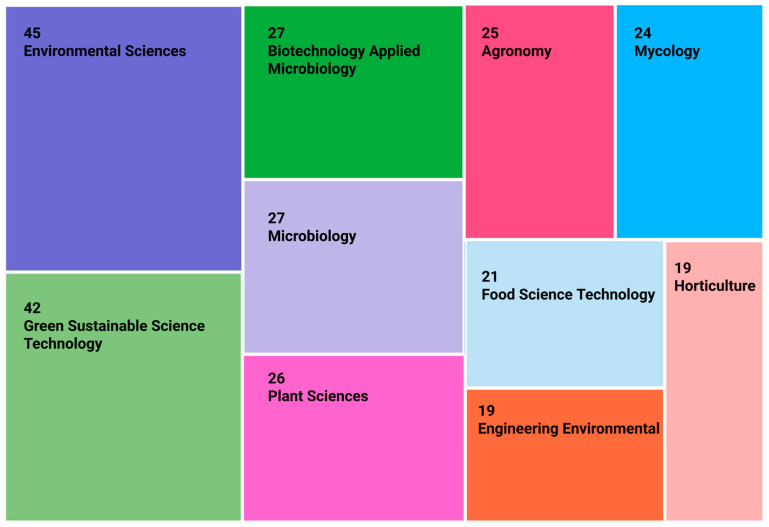
Treemap showing the subject categories of mushroom-related publications (RStudio bibliometric tools).

The disciplinary distribution of publications (Figure 3) demonstrates the multidisciplinary nature of mushroom research. Most studies fall within Environmental Sciences, Green and Sustainable Science and Technology, Biotechnology and Applied Microbiology, and Microbiology, followed by Plant Sciences, Agronomy, Mycology, and Food Science and Technology. This spread reflects the integration of environmental, agricultural, and microbial perspectives within the field [11].

**Figure 4:**
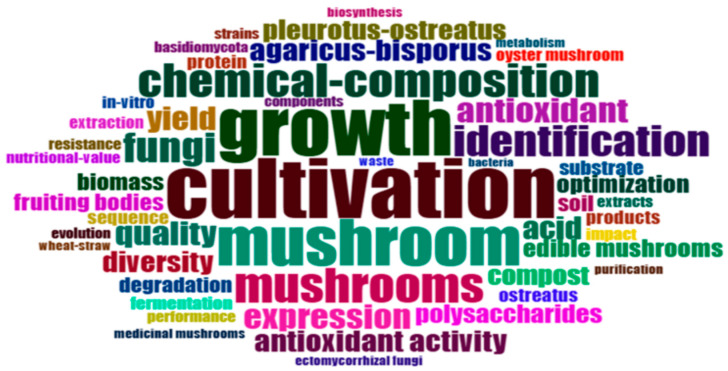
Word cloud of frequently used keywords in mushroom research (RStudio bibliometric tools).

The word cloud presented in Figure 4 highlights frequently used keywords such as cultivation, growth, mushroom, and chemical composition, indicating that much of the current literature focuses on improving yield and optimizing substrate use. In contrast, terms associated with molecular-level research, including gene expression, transcriptome, and CRISPR, appear infrequently. This suggests that while molecular and health-related topics are emerging, they remain secondary to production-focused studies, a pattern also noted in broader sustainability and agricultural biotechnology research [12].

**Figure 5:**
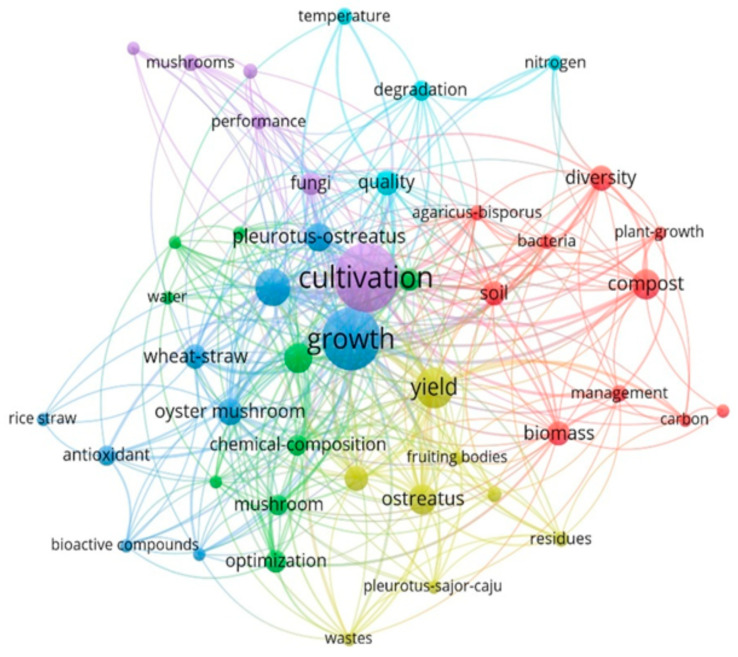
Keyword co-occurrence network showing three main research clusters: cultivation and productivity, substrate and compound analysis, and microbial or compost-related studies (VOSviewer).

Keyword co-occurrence analysis (Figure 5) further supports this observation. Three dominant clusters are evident: cultivation and productivity, substrate use and chemical composition, and environmental or microbial aspects of mushroom systems. Molecular biology-related terms are weakly connected and positioned at the periphery of the network, indicating limited integration of advanced molecular tools within applied mushroom research [12].

The weak representation and connectivity of terms such as CRISPR, transcriptomics, gene expression, artificial intelligence, and machine learning provided a clear rationale for the focus of this review. These underexplored yet potentially transformative areas informed the emphasis on molecular approaches, microbiome studies, and precision cultivation strategies discussed in subsequent sections [13].

**Figure 6:**
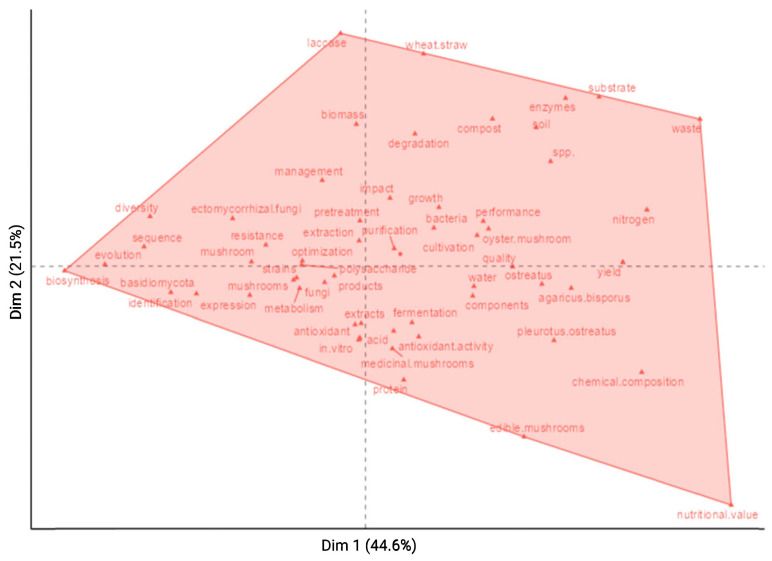
Factorial analysis (MCA plot) showing central and peripheral themes in mushroom research (RStudio bibliometric tools).

To further examine thematic relationships, Multiple Correspondence Analysis (MCA) was performed based on keyword co-occurrence (Figure 6). Productivity-related terms occupy the central region of the plot, whereas sustainability- and molecular-related terms form distinct peripheral clusters. This separation indicates that molecular and omics-based studies are still largely disconnected from mainstream sustainability-driven mushroom research, highlighting the need for integrative approaches that link molecular insights with practical production systems [14].

The bibliometric analysis was used not only to map publication trends but also to identify gaps that shape the structure of this review. While cultivation and substrate optimization dominate the literature, molecular regulation, microbiome interactions, and climate-adapted production remain weakly represented and poorly connected to sustainability-focused studies. For this reason, the following sections focus on molecular mechanisms, symbiotic interactions, and integrative strategies that can improve mushroom production under resource- and climate-limited conditions.

## 3. Adaptations of Mushrooms to Diverse Climates and Ecosystems

The bibliometric analysis highlights significant research trends and knowledge gaps in understanding how mushrooms adapt to diverse ecosystems. Tropical and arid regions have attracted considerable attention due to their agricultural and ecological significance, while temperate and boreal mushrooms remain essential contributors to nutrient cycling and forest ecosystem stability. These varied climatic regions reveal the influence of environmental pressures on fungal physiology, enzymatic activity, and ecological roles. Insights into these adaptations offer valuable opportunities to optimize sustainable mushroom cultivation practices tailored to specific environmental challenges [15]. Table 1 provides a detailed overview of selected mushroom species across different climates, detailing their habitat, edibility, and key adaptive traits. Species included in Table 1 were selected based on documented occurrence in distinct climatic zones, availability of ecological or physiological adaptation data, and relevance to food, medicinal, or ecosystem functions.

### 3.1. Tropical Mushrooms

Tropical mushrooms, including *Volvariella volvacea* (straw mushroom) [50] and *Lentinula edodes* (shiitake) [32], are well-adapted to the warm, humid climates where they play a crucial role in decomposing lignocellulosic substrates into essential soil nutrients. Their rapid growth cycles and metabolic versatility allow them to exploit a wide range of substrates, from agricultural residues to decaying wood and leaf litter [19].

A key adaptation of tropical mushrooms is their ability to retain moisture using specialized hyphal structures, which prevent desiccation during brief dry periods [20]. These characteristic supports sustained metabolic activity despite fluctuating environmental conditions. Additionally, species like *Pleurotus ostreatus* (oyster mushroom) efficiently degrade lignin and cellulose, making them valuable for biotechnological applications, including agricultural waste recycling. Some tropical species also exhibit thermotolerance, enabling them to survive in elevated temperatures [21].

### 3.2. Arid Mushrooms

Mushrooms in arid regions such as *Terfezia* sp. (desert truffle) and *Podaxis pistillaris* have evolved xerophytic traits that allow them to survive in water-scarce and nutrient-poor environments [34,42]. Among these, the Terfezia–Helianthemum symbiosis represents a promising but understudied model for sustainable cultivation in arid ecosystems, offering insights into native fungal–plant resilience. These fungi are particularly noted for their ability to form ectomycorrhizal associations with host plants such as Helianthemum spp., which enhance nutrient and moisture acquisition in arid soils [51]. A defining adaptation of arid mushrooms, including desert truffles, is the production of osmoprotectants such as trehalose and mannitol, which help mitigate dehydration and thermal stress. In addition, heat-shock proteins (HSPs) contribute to cellular stability and protection against oxidative damage under extreme conditions [35].

*Terfezia boudieri* contributes to soil stabilization and enhances plant productivity through its symbiotic relationship with *Helianthemum* spp. [52]. Similarly, *Podaxis pistillaris* thrives in desert environments by utilizing elongated stems and splitting caps to efficiently disperse spores under dry conditions [42]. *Schizophyllum commune* also exhibits notable resilience and moisture retention, allowing it to adapt to both decaying wood and arid habitats [44]. Species adapted to these environments exhibit traits including efficient water retention, production of osmoprotective compounds, and strong dependence on plant–fungal symbioses. Desert truffles, for example, rely on ectomycorrhizal associations to enhance water and nutrient uptake under drought conditions, while other arid-adapted fungi display structural and physiological traits that support spore dispersal and persistence in dry habitats. Together, these adaptations highlight the potential of native and desert-associated mushrooms as models for sustainable cultivation in arid regions, including the Middle East [44].

### 3.3. Temperate and Boreal Mushrooms

Mushrooms in temperate and boreal climates sustain forest ecosystems by adapting to seasonal variations, nutrient limitations, and temperature extremes through symbiosis, decomposition, and carbon cycling. Ectomycorrhizal fungi like *Amanita muscaria* and *Boletus edulis* form mutualistic associations with trees, enhancing nitrogen and phosphorus uptake in nutrient-poor soils while receiving carbohydrates in return, supporting both tree survival and forest biodiversity [18,23]. Many temperate and boreal mushrooms, such as *Pleurotus ostreatus* and *Armillaria* spp., function as saprotrophs, decomposing lignocellulosic materials and recycling nutrients to maintain soil fertility and sustain nutrient cycling [38]. Cold-adapted boreal mushrooms such as *Mortierella* and *Mrakia* is produce antifreeze proteins and cryoprotectants to stabilize cell membranes, prevent ice formation, and sustain nutrient cycling even in subzero temperatures. Some boreal fungi also develop extracellular polysaccharides for moisture retention and frost resistance [39]. Boreal fungi also contribute to carbon cycling, as species like *Suillus luteus* regulate soil carbon dynamics by decomposing organic matter [45]. Some temperate species, such as *Morchella esculenta* [34] (morel) and *Tricholoma matsutake* [42] (matsutake), are commercially valuable due to their culinary and medicinal properties.

## 4. Symbiotic and Ecological Interactions in Mushrooms

The bibliometric analysis highlights key research on the enzymatic capabilities and ecological roles of mushrooms, particularly in lignocellulose degradation and ecosystem sustainability. Beyond these functions, mushrooms play crucial roles in symbiotic interactions, nutrient cycling, and soil health. These relationships, including mycorrhizal associations and microbial interactions, enable mushrooms to thrive in diverse environments while supporting plant productivity and ecosystem resilience.

### 4.1. Mycorrhizal Associations with Host Plants

Mushrooms form essential mycorrhizal associations with host plants, facilitating nutrient exchange, particularly in nutrient-deficient soils and arid environments. Fungi provide plants with vital minerals such as phosphorus and nitrogen, while plants reciprocate by supplying carbohydrates produced through photosynthesis. This exchange strengthens resilience and enhances plant growth under challenging conditions [43].

**Figure 7:**
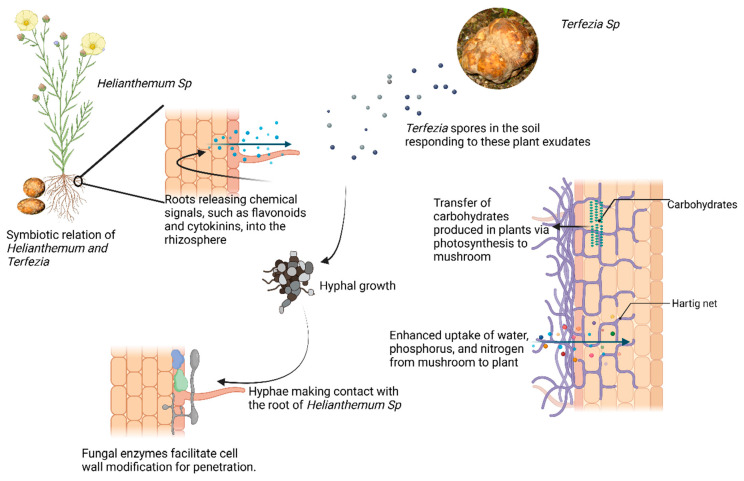
Schematic representation of the symbiotic interaction between *Terfezia* species (desert truffles) and *Helianthemum* plants (created in Biorender, Francis, D. V. (2026) https://BioRender.com/bo4z2g2 (accessed on 10 January 2026)).

The ectomycorrhizal relationship between desert truffles (*Terfezia* spp.) and *Helianthemum* species exemplifies this ecological partnership. This example is included due to its ecological relevance in arid environments and its potential as a model for sustainable desert truffle cultivation in climate-stressed regions. This interaction begins when *Helianthemum* roots exude chemical signals, including flavonoids, into the rhizosphere, which act as chemo-attractants to nearby fungal spores. These signals trigger the germination of *Terfezia* spores, initiating the pre-symbiotic phase. During this stage, the fungal hyphae elongate and grows toward the roots, driven by the gradient of exuded flavonoids. Concurrently, the fungus upregulates genes responsible for membrane synthesis and secretion, preparing for colonization [51]. Upon contact, the hyphae adhere to the root surface and form a fungal sheath around the root tips. This sheath acts as a protective barrier and facilitates nutrient exchange. As the colonization progresses, fungal hyphae penetrate the root cortex without breaching plant cells, forming a dense network known as the Hartig net [45]. As depicted in Figure 7, this network serves as the primary site for nutrient transfer between the fungal hyphae and root cells.

Fungal-derived auxins, such as indole-3-acetic acid (IAA), play a crucial role in modifying root architecture during these interactions. IAA suppresses taproot elongation while promoting the development of lateral and secondary roots, increasing the root system’s surface area and improving nutrient uptake. Additionally, cytokinins produced by the plant regulate these morphological changes, ensuring a balanced development of fungal and root structures [46].

The symbiotic relationship is further supported by nutrient transporter genes expressed in both partners. The fungus upregulates phosphate and nitrogen transporter genes, enabling efficient absorption and delivery of these minerals to the plant. Simultaneously, the plant expresses carbohydrate transporter genes to supply photosynthetic products to the fungus, completing the reciprocal nutrient exchange [47].

At the molecular level, sugar signaling pathways play a pivotal role in establishing and maintaining this symbiosis. Carbohydrates released by the host plant act as both a nutrient source and signaling molecules that regulate fungal growth and symbiotic gene expression. Sugar availability influences fungal gene networks related to hyphal elongation and nutrient transporter activity, ensuring a synchronized and efficient interaction between the partners. These sugar signals also modulate the production of fungal auxins, further enhancing root modification and colonization efficiency [48].

The associated microbiome also plays a critical role in sustaining this symbiosis. Specific bacterial communities enhance nutrient availability and promote fungal fruiting body development. In arid regions like the Negev Desert, *Terfezia boudieri* forms symbioses with *Helianthemum sessiliflorum*, improving plant physiological functions, including photosynthesis and transpiration. Similarly, desert truffle species such as *Terfezia* and *Tirmania* form symbiotic associations with *Helianthemum* species across arid regions of the Arabian peninsula, contributing to ecosystem stability and plant–soil interactions under desert conditions [51].

While desert truffle–Helianthemum symbioses emphasize drought resilience and nutrient scavenging under arid conditions, ectomycorrhizal associations in temperate and boreal systems primarily support carbon exchange and long-term forest nutrient cycling, highlighting climate-specific functional specialization of mycorrhizal interactions.

### 4.2. Lignin Degradation: Genetic and Molecular Insights

Bibliometric analysis highlights substrate optimization as a key focus in sustainable mushroom cultivation, particularly in utilizing lignocellulosic biomass. Lignin, a complex and recalcitrant polymer, restricts nutrient availability, making its degradation essential for efficient substrate utilization and improved sustainability in mushroom farming.

#### 4.2.1. Enzymatic Pathways for Lignin Degradation

**Figure 8:**
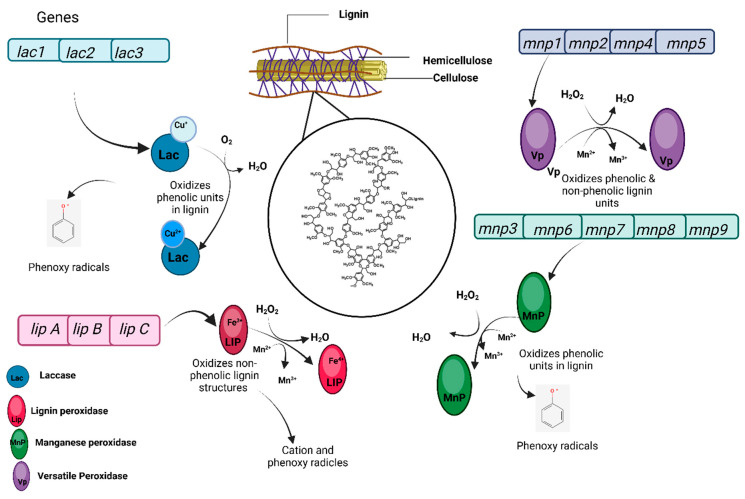
Diagrammatic representation of enzymatic lignin degradation pathways in *Pleurotus ostreatus* (Biorender Created in BioRender. Francis, D. V. (2026) https://BioRender.com/5ss8lxk (accessed on 10 January 2026)).

Mushrooms degrade lignin through an advanced enzymatic system comprising oxidative enzymes such as laccases, manganese peroxidases (MnPs), lignin peroxidases (LiPs), and auxiliary enzymes like lytic polysaccharide monooxygenases (LPMOs) [50], as shown in Figure 8.

Laccases, encoded by genes like *Lac1*, *Lac2*, and *Lac3* in *Pleurotus ostreatus*, catalyze lignin oxidation through radical-mediated mechanisms. These radicals destabilize the lignin polymer, allowing subsequent enzymatic attacks. Laccase expression is regulated by environmental factors like nitrogen depletion and oxidative stress, ensuring optimal activity under resource-limited conditions [53].

MnPs oxidize Mn^2+^ to Mn^3+^ in the presence of hydrogen peroxide (H_2_O_2_), generating reactive ions that penetrate lignin’s complex matrix and target phenolic structures. In *P. ostreatus*, the *mnp* gene family (*mnp1*–*mnp9*) is crucial, with *mnp3* exhibiting high activity in Mn^2+^-enriched environments [54]. Versatile peroxidases (VPs), encoded by *mnp4*, further extend lignin degradation by acting on both phenolic and non-phenolic lignin substrates, increasing degradation efficiency [55].

LPMOs from the AA9 family enhance substrate accessibility by cleaving cellulose-lignin linkages, facilitating better enzymatic action by laccases and peroxidases. This synergistic interaction is vital for deconstructing tightly bound lignocellulosic complexes into smaller, more accessible units, optimizing nutrient release for fungal metabolism [56].

#### 4.2.2. Genetic and Molecular Regulation

The expression and activity of ligninolytic enzymes are regulated by complex genetic and molecular networks that dynamically respond to environmental stimuli. Transcription factors, such as C6 zinc-finger proteins, serve as crucial regulators of genes encoding laccases and MnPs [57]. These transcription factors sense changes in external conditions, including nutrient availability and oxidative stress, and modulate gene expression accordingly. Nutrient limitation often triggers the upregulation of laccase and MnP genes, thereby enhancing the fungus’s ability to degrade lignin and access nutrients. Signal transduction pathways further fine-tune these regulatory responses. The MAPK signaling cascade is particularly activated under oxidative stress, promoting the transcription of ligninolytic genes. Similarly, the cAMP signaling pathway plays a pivotal role in sensing nutrient levels and coordinating enzyme production, ensuring that ligninolytic activity is optimally aligned with environmental conditions [58]. Epigenetic modifications, such as histone acetylation and DNA methylation, add another layer of regulatory control by altering chromatin structure [59]. These modifications regulate the accessibility of transcriptional machinery to target genes, enabling fungi to adapt quickly to changing environmental conditions and ensuring efficient lignin degradation across diverse substrates.

#### 4.2.3. Technological Innovations in Lignin Degradation

Advancements in molecular biology have refined lignin degradation research in *Pleurotus ostreatus*. CRISPR/Cas9 genome editing has precisely modified lignin-modifying enzymes (*vp2*, *vp3*, *mnp3*), confirming their roles. Multi-gene editing using polycistronic tRNA and guide RNA constructs has enhanced enzymatic efficiency and reduced functional redundancies. Marker-free genome editing, which avoids foreign DNA, has further optimized fungal strains while addressing biosafety concerns [60].

RNA interference (RNAi) has been applied to suppress genes in competing metabolic pathways, thereby redirecting fungal metabolic resources toward lignin degradation. This approach has enhanced ligninolytic enzyme production and activity in industrial fungal strains. High-frequency gene targeting using ku80-deleted strains has facilitated homologous recombination, enabling precise genetic manipulations to elucidate gene functions associated with lignin degradation [61].

Transcriptomic analyses have identified transcription factors such as Wtr1 and Gat1, which regulate ligninolytic gene expression. CRISPR/Cas9-mediated knockouts of these genes have demonstrated their essential roles in modulating ligninolytic pathways [62]. Proteomic studies have identified post-translational modifications, such as phosphorylation and glycosylation, that regulate enzyme activity, stability, and secretion, influencing the efficiency of ligninolytic enzymes like laccases and manganese peroxidases. Advanced techniques, including mass spectrometry, have clarified these modifications’ roles in lignin degradation. Complementing this, metabolomic analyses have revealed key intermediates and pathways in lignin breakdown, emphasizing the interaction between metabolic processes that generate essential mediators like hydrogen peroxide. Combined proteomic and metabolomic insights have advanced the understanding of how environmental factors regulate the specificity and efficiency of ligninolytic systems [60].

The rhizosphere microbiome, a diverse microbial community surrounding the plant-fungal interface, enhances mushroom ecology by influencing nutrient availability, stress resilience, and productivity. Beneficial microbes such as *Pseudomonas putida* and *Bacillus subtilis* solubilize phosphorus and fix nitrogen, improving soil fertility, especially in arid regions [63]. In Terfezia–Helianthemum symbioses, the mycorrhizal association improves nutrient exchange and plant fitness under arid conditions [52], while associated microbial communities further influence hyphal development and fruiting dynamics [51].

Microbial diversity also suppresses soil pathogens like *Trichoderma* spp. through competitive exclusion and antimicrobial compound production, as seen in antibiotic-secreting *Streptomyces* species. Additionally, microbes mitigate abiotic stresses like salinity and drought by producing osmoprotectants (e.g., proline, glycine betaine) and detoxifying enzymes (e.g., dehalogenases, peroxidases). Rhizospheric microbes further stimulate fungal and plant growth via phytohormones such as indole-3-acetic acid (IAA), gibberellins, and cytokinins, which regulate root modifications and enhance fungal auxin production in *Terfezia-Helianthemum* systems. Quorum-sensing compounds coordinate microbial interactions, optimizing cooperative behavior with fungi [46].

Integrating beneficial microbes into mushroom cultivation enhances productivity and sustainability. Microbial inoculants tailored to specific fungal species, such as *Agaricus bisporus* co-cultivated with *Pseudomonas fluorescens* and *Bacillus amyloliquefaciens*, improve lignocellulose degradation, reduce contamination, and enhance nutrient availability. Microbiome engineering also strengthens stress-resilient cultivation systems by selecting drought- and salinity-tolerant microbial strains. Additionally, microbial communities influence fungal fruiting body formation by producing signaling molecules that trigger hormonal pathways, as observed in *Flammulina velutipes*, where auxin-like compounds stimulate fruiting body initiation [64].

Metagenomic studies on *Ganoderma lucidum* and *Agaricus bisporus* have identified microbial genes involved in synthesizing plant growth regulators, underscoring the microbiome’s crucial role in fungal reproduction. These microbial interactions provide innovative strategies for optimizing substrate utilization, improving resilience, and enhancing mushroom farming productivity [65].

Comparative analyses across white-rot fungi indicate that although ligninolytic enzyme families are conserved, their regulation, expression intensity, and synergistic deployment vary with substrate type, environmental stress, and cultivation context.

Collectively, these studies demonstrate that mushroom ecology is governed by tightly coupled interactions among symbiosis, enzymatic capacity, and associated microbiomes, rather than by isolated mechanisms. While arid-adapted systems prioritize stress tolerance and efficient nutrient acquisition, temperate and boreal mushrooms emphasize sustained carbon cycling and ecosystem stability. Integrating these ecological strategies with molecular and microbiome-level insights provides a framework for designing climate-resilient and sustainable mushroom cultivation systems.

## 5. Integrative Omics Approaches in Mushroom Cultivation

Advancements in omics technologies have transformed our understanding of mushroom biology and opened new opportunities to improve cultivation practices. Approaches such as transcriptomics, proteomics, genomics, synthetic biology, and metagenomics have been used to optimize substrate utilization, enhance stress tolerance, and improve yield quality. While these tools have generated valuable insights into fungal growth and metabolism, they are often applied independently. Transcriptomic studies primarily describe gene expression changes in response to cultivation conditions and environmental stress, whereas proteomic analyses identify enzymes and proteins directly involved in substrate degradation and nutrient uptake. Genomic studies define the genetic potential of different species and strains, helping explain variation in growth performance and resilience. Together, these findings indicate that effective improvement of mushroom cultivation depends on coordinated regulation across multiple molecular levels, although such integration remains limited in current studies.

These omics approaches specifically address key gaps in conventional mushroom cultivation, including limited understanding of stress tolerance mechanisms, inefficient substrate conversion, strain-specific yield variability, and inconsistent disease resistance under changing environmental conditions.

### 5.1. Transcriptomics and Proteomics

Transcriptomic studies have been pivotal in understanding stress responses in mushrooms. Analyses of *Pleurotus giganteus* under high-temperature stress revealed the upregulation of genes associated with heat shock proteins (HSPs), protein kinases, and transcription factors, highlighting their roles in thermal resilience [66]. In *Pleurotus tuoliensis*, RNA-seq analysis under prolonged heat stress identified differentially expressed genes (DEGs) involved in ergosterol biosynthesis, small molecule biosynthesis, and amino acid metabolism, offering insights into the mechanisms underpinning thermotolerance [67].

Proteomic investigations in *Lentinula edodes* (shiitake mushroom) identified key nutrient transporters such as ammonium and phosphate transporters, crucial for efficient nutrient assimilation and enhanced substrate utilization. In response to heat stress, the upregulation of heat shock proteins (HSPs), including Hsp40, Hsp70, and Hsp98, was evident, demonstrating their protective role in maintaining cellular integrity under stress conditions. Additionally, metabolic pathways linked to indoleacetic acid (IAA) biosynthesis were significantly activated, with enzymes such as indole-3-pyruvate monooxygenase (LeYUCCA) being upregulated [68]. These adaptations highlight how proteomic changes help improve stress tolerance and support growth under different environmental conditions. Further research on *Flammulina velutipes* emphasizes how cold stress prompts enzymatic adaptations tailored to substrate-specific requirements, enhancing lignocellulose degradation efficiency [69].

Combined transcriptomic and proteomic approaches have shed light on disease resistance mechanisms. Comparative transcriptomic studies of resistant and sensitive strains of *P. ostreatus* during *Pseudomonas tolaasii*-induced Brown blotch disease (BBD) identified DEGs related to oxidation-reduction enzymes, cell wall integrity, and signal transduction pathways, revealing the molecular underpinnings of resistance [70]. Such integrative analyses have proven invaluable in identifying candidate genes for breeding disease-resistant strains and developing targeted interventions for pathogen control.

These combined efforts in transcriptomics and proteomics not only enhance our understanding of stress responses and nutrient utilization but also pave the way for innovations in mushroom cultivation, such as breeding thermotolerant and disease-resistant strains, optimizing substrates, and improving yield quality under diverse environmental conditions.

Across species, transcriptomic and proteomic studies consistently reveal that stress adaptation, nutrient uptake efficiency, and disease resistance are regulated through conserved yet context-dependent molecular pathways, highlighting shared adaptive strategies despite species-specific expression patterns.

### 5.2. Synthetic Biology

Synthetic biology enables the modification of fungal metabolic pathways to enhance metabolite production and stress resilience. Research in *Ganoderma lucidum* has shown that the transcription factor GCN4 controls the mitochondrial pyruvate carrier (MPC) under nitrogen-limited conditions by directly binding to its promoter. This regulation enhances the TCA cycle and the production of secondary metabolites like ganoderic acids. The role of MPC in regulating carbon flux and secondary metabolism in *G. lucidum* under nitrogen-limited conditions underscores its potential for synthetic biology applications. Silencing GlMPC modifies pyruvate transport and metabolic pathways, significantly increasing secondary metabolite production [71]. In *Aspergillus oryzae*, the absence of MPC shifts carbon metabolism, leading to increased production of metabolites such as lactic acid and 2,3-butanediol [72]. Similar strategies can be utilized in other fungal species, including *Pleurotus ostreatus*, to optimize the production of valuable compounds.

### 5.3. Metagenomic Approaches

Metagenomics has transformed our understanding of microbial communities in mushroom cultivation by analyzing genetic material directly from environmental samples. Studies on *Agaricus bisporus* have identified bacteria such as *Pseudomonas putida* that enhance lignin degradation, improving nutrient availability and increasing yields. Additionally, this approach reveals microbial antagonists like *Trichoderma* species, enabling strategies to suppress contaminants through beneficial microbes or altered cultivation practices [73].

Research on wild mushrooms in Indian tropical and temperate forests highlights nitrogen-fixing bacteria such as *Bradyrhizobium* species, which enhance ectomycorrhizal associations, promoting mushroom growth [74]. In *Agaricus bisporus* compost, bacteria like *Thermobifida* and *Thermostaphylospora* synergize with fungal mycelia to degrade lignocellulosic substrates, boosting nutrient release and substrate utilization [75]. Metagenomics has also identified bacterial and fungal roles in hormonal signaling pathways that regulate fruiting body development. These findings demonstrate the potential of metagenomics to unravel microbial roles in optimizing substrate use, improving mushroom yields, and enhancing cultivation practices.

### 5.4. Genomics and Metabolomics

To complement transcriptomic and proteomic studies, genomics and metabolomics provide deeper insights into mushroom biology. Genomics has transformed mushroom research by enabling the identification of genetic traits linked to stress resistance, enzymatic activity, and bioactive compound production. Sequencing the genomes of species such as *Ganoderma lucidum* [76] and *Pleurotus eryngii* has revealed the genes responsible for secondary metabolite biosynthesis, including triterpenoids and polysaccharides, which have medicinal and therapeutic applications [77]. Advances in genome mining have also facilitated the discovery of gene clusters involved in the production of bioactive compounds in *Laetiporus sulphureus* and other edible fungi, supporting synthetic biology and strain improvement initiatives [78].

Metabolomics involves the comprehensive profiling of small molecules within cells, focusing on metabolic pathways and their regulation. Research on the metabolome of *Ganoderma lucidum* has identified bioactive compounds like triterpenoids and polysaccharides with therapeutic applications [79]. Similarly, untargeted metabolomics of edible mushroom by-products, such as those from *Hypsizygus marmoreus*, revealed dynamic changes in antioxidant capacity and non-volatile metabolites, particularly flavonoids and amino acid derivatives, during fermentation [80]. These findings highlight the nutritional enhancement achievable through tailored processing. Metabolomic profiling of *Tricholoma mesoamericanum* has revealed the presence of 181 metabolites, including terpenoids, glycerophospholipids, and amino acids, which contribute to its antioxidant properties and bioactive potential [81]. Metabolomic techniques in culinary mushrooms have enhanced understanding of how metabolites are influenced by factors such as genetic variations, post-harvest handling, and processing methods [82]. These studies highlight the role of multiomics in optimizing the quality of mushrooms, focusing on their flavor, nutritional composition, and bioactive compound retention.

Taken together, the studies discussed above point toward an integrated production model in which mycorrhizal associations, microbial communities, and molecular regulation act as interconnected components of mushroom cultivation systems. Mycorrhizal and microbiome interactions influence nutrient availability and stress buffering at the substrate level, while molecular regulation governs fungal responses through gene expression and metabolic adjustment. Environmental factors such as temperature, moisture, and substrate composition further shape these interactions, collectively determining productivity, resilience, and sustainability, particularly in resource-limited environments.

Despite these promising advances, several limitations remain. Molecular responses are often strain- or species-specific, limiting broad generalization, and improvements observed under controlled laboratory conditions do not always translate to large-scale cultivation. Variability in experimental design, substrates, and growth conditions complicates cross-study comparisons, while costs, technical expertise requirements, regulatory approval processes for genetically modified strains, and public acceptance issues may restrict industrial adoption. These challenges underscore the need for cautious interpretation and validation of multi-omics strategies under realistic cultivation and regulatory frameworks.

## 6. Sustainable Mushroom Production

Sustainable mushroom production integrates ecological principles and technology to reduce environmental impact while ensuring economic viability. By utilizing renewable resources and circular economic strategies, mushroom farming minimizes waste, conserves energy, and supports global food security. The circular economy model repurposes agricultural by-products such as wheat straw, sawdust, and spent coffee grounds as substrates for mushrooms like *Pleurotus ostreatus* [21]. These materials are converted into food, while spent mushroom substrate (SMS) improves soil fertility, water retention, and microbial diversity [6].

**Figure 9:**
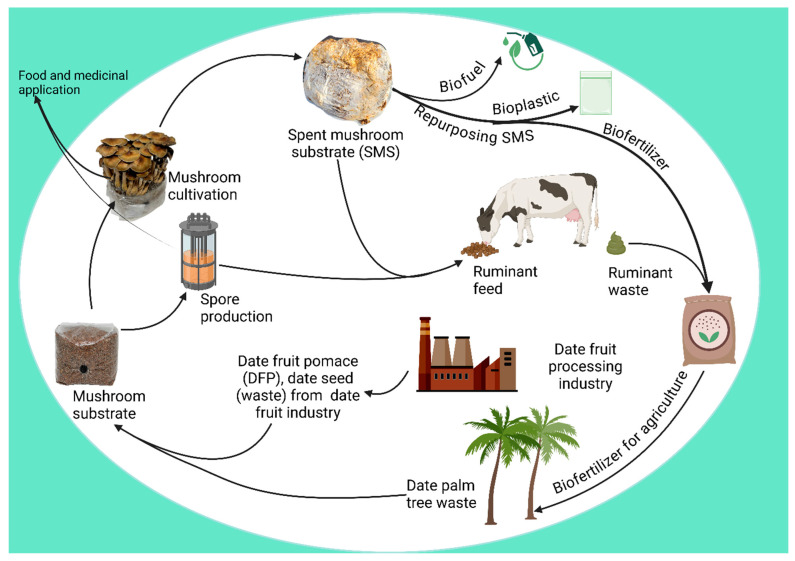
Circular economy model for mushroom production in arid regions, repurposing date palm waste for sustainable cultivation and resource recycling (Biorender Created in BioRender. Francis, D. V. (2026) https://BioRender.com/oxdmhye (accessed on 10 January 2026)).

In arid regions such as the Middle East, where the date palm industry generates large amounts of agricultural waste, date palm residues are repurposed for mushroom cultivation, supporting food production and sustainable agriculture. Mushroom-based systems also improve soil stabilization, biodiversity conservation, and carbon sequestration, contributing to land restoration [83]. Figure 9 illustrates the circular economy model for mushroom cultivation in arid environments, demonstrating how date palm waste is efficiently utilized to create a self-sustaining system.

Low-cost technologies make mushroom farming viable in resource-limited regions. Solar-powered systems regulate optimal temperature and humidity, reducing energy use. IoT-based monitoring improves efficiency, and hydroponic methods adapted for mushroom farming enable production in arid climates through soilless techniques and passive solar heating [84].

AI, machine learning (ML), and nanotechnology are increasingly explored to improve mushroom cultivation efficiency [85]. AI-based tools help detect contamination risks and optimize substrate use, while nanotechnology supports nutrient delivery and moisture retention, which is particularly relevant for water-limited environments [86,87].

Beyond environmental considerations, mushroom farming also supports economic value creation through products such as medicinal mushrooms (*Ganoderma lucidum*, *Lentinula edodes*) and bioactive compounds for nutraceutical applications. Spent mushroom substrate is further reused for biofertilizers and biogas production, supporting full resource utilization within circular production systems. Together, these approaches demonstrate how sustainability principles are implemented in mushroom cultivation through circular resource use and targeted technological support [6].

## 7. Challenges and Future Directions

Mushroom cultivation faces key challenges in achieving environmental sustainability while maintaining economic viability, particularly for smallholder farmers. Large-scale production requires substantial inputs of water, energy, and substrate materials, while smaller operations often lack access to essential technologies, financing, and training. Climate variability can disrupt crop cycles, and inconsistent substrate availability may limit continuous production. In addition, intensive cultivation practices can contribute to methane emissions during anaerobic composting and nutrient runoff from spent mushroom substrate, potentially affecting soil and water quality [6].

While synthetic biology and CRISPR/Cas9 approaches offer opportunities to improve yield performance and lignocellulose degradation, their adoption remains limited by regulatory constraints and public concerns related to genetically modified organisms, particularly in regions with strict biosafety regulations [60]. Circular economy models based on agricultural waste recycling enhance sustainability, but the infrastructure required for effective bioconversion is often inaccessible to small-scale producers, reinforcing inequalities in access and benefit sharing [88].

To address these challenges, coordinated efforts across policy, research, and industry are required. Advances in nanotechnology, epigenetics, and AI-based monitoring systems have the potential to improve substrate performance, stress tolerance, and resource-use efficiency. Epigenetic mechanisms in species such as *Pleurotus ostreatus* and *Ganoderma lucidum* play roles in stress regulation and bioactive compound production, contributing to crop resilience [19]. Similarly, recent molecular studies on ectomycorrhizal fungi such as *Sarcodon* spp. have revealed diverse secondary metabolites with ecological and functional relevance, highlighting the need for deeper exploration of biosynthetic pathways under environmental stress [89]. Nanotechnology-based approaches may support nutrient delivery and reduce dependence on chemical inputs, although further validation is needed.

AI and data analytics tools are being explored to optimize environmental control, monitor contamination risks, and predict yields in controlled cultivation systems. While these tools show promise in reducing operational costs and improving disease management, their application remains limited in many production settings and require adaptation to local conditions [90].

Equitable policy frameworks that promote knowledge exchange, technology transfer, and inclusive financing are essential for supporting under-resourced growers. Strengthening infrastructure and training at the community level, particularly in rural and arid regions, will be critical for integrating advanced technologies with locally adapted cultivation practices. Aligning innovation with regional needs and practical feasibility will be key to achieving resilient and sustainable mushroom production systems [91].

## 8. Conclusions

This review combines bibliometric analysis with ecological and molecular evidence to show how mushroom research is currently shaped and where important gaps remain. While most studies focus on cultivation methods and yield, fewer works connect these outcomes with molecular regulation, microbiome function, or climate adaptation. This disconnect is especially clear for arid and semi-arid regions, where environmental stress strongly affects production but remains underexplored.

Future research should focus on linking molecular responses directly to cultivation performance. This includes identifying which stress-response pathways, enzyme systems, and microbial interactions consistently improve substrate conversion, yield stability, and resistance to heat and water stress. More emphasis is also needed on validating these findings across different strains, substrates, and growing conditions to improve reproducibility and practical relevance.

From an application perspective, progress will depend on simple, scalable approaches that fit local conditions. Using region-specific agricultural waste, improving basic environmental control, and integrating low-cost monitoring tools can help translate research advances into practice. With better alignment between molecular insight and cultivation needs, mushroom production can play a stronger role in sustainable food systems, particularly in climate-challenged regions.

## Figures and Tables

**Table 1:** Habitat, edibility, and key traits of mushrooms across various climates and ecosystems.

Species	Common Name	Habitat	Edibility	Adaptation Characteristics
*Agaricus columellatus*	Minor pouch Agaricus	Deserts of the southwestern United States,	Unknown	Pouch-like basidiocarp; reduced exposure in arid habitats [16].
*Agaricus deserticola*	Gasteroid Agaricus	Southwestern and western North America, arid and semi-arid habitats	Unknown	Secotioid morphology; reduced cap opening [15].
*Agrocybe aegerita*	Black poplar mushroom	Grows on hardwoods, especially poplars	Edible	Wood-decay specialization; rapid substrate colonization [17].
*Amanita muscaria*	Fly Agaric	Coniferous and deciduous forests	Inedible	Ectomycorrhizal symbiosis; secondary metabolite production [18].
*Armillaria* spp.	Honey fungus	Deciduous and coniferous forests	Edible	Rhizomorph formation; long-distance nutrient transport [19].
*Auricularia auricula*	Wood ear mushroom	Moist, shaded environments.	Edible	Gelatinous basidiocarp; desiccation tolerance [20].
*Auricularia polytricha*	Cloud ear mushroom	Warm, humid climates, grows on dead or decaying wood	Edible	Moisture retention via jelly-like tissue [21].
*Battarrea phalloides*	Sandy stiltball	Dry, sandy soils, found in Europe, Africa, Australia, Asia, and the Americas	Inedible	Elevated stipe; wind-assisted spore dispersal [22].
*Boletus edulis*	Porcini	Temperate forests, under oak, pine	Edible	Ectomycorrhizal association; forest nutrient exchange [23].
*Calvatia gigantea*	Western giant puffball	Grasslands and open areas in arid regions	Edible when young	Puffball sporulation; rapid spore release when mature [24].
*Cantharellus cibarius*	Golden chanterelle	Temperate forests, mossy areas	Edible	Mycorrhizal lifestyle; soil moisture dependence [25].
*Cercopemyces crocodilinus*	-	Arid regions; associated with mountain mahogany	Unknown	Secotioid development; adaptation to dry soils [26].
*Clavulina cristata*	Crested coral fungus	Deciduous and coniferous forests	Edible	Mycorrhizal growth; coral-like branching morphology [27].
*Cynomorium coccineum*	Desert thumb	Deserts of the Mediterranean and Arabian Peninsula	Inedible	Holoparasitism; reduced fungal structures [28].
***Gyrophragmium californicum***	-	Deserts of North America	Inedible	Secotioid form; protection against desiccation [29].
*Hygrophorus camarophyllus*	Smoky gilled waxcap	Boreal and temperate forests	Edible	Cold tolerance; waxy cuticle [30].
*Lactarius deterrimus*	False saffron milkcap	Boreal and temperate forests	Edible	Latex production; mycorrhizal nutrient exchange [31]
*Lentinula edodes*	Shiitake	Native East Asia, grows on decaying hardwood trees	Edible	Hardwood specialization; efficient lignocellulose degradation [32].
*Montagnea arenaria*	Namaqua black cap	Arid regions, often found in sandy soils	Unknown	Woody stipe; arid-environment persistence [33].
*Montagnea haussknechtii*	Small spored Namaqua black cap	Dry sandy soils, grasslands, and savannas	Unknown	Reduced spores; structural stability in dry soil [33].
*Morchella esculenta*	Morel	Temperate regions, often on sandy soils	Edible	Dual saprotrophic–symbiotic lifestyle [34].
*Mycenastrum corium*	Corkstar puffball	Arid and semi-arid regions of Australia and the southwestern United States	Unknown	Thick peridium; delayed spore release [35].
*Phellorinia herculeana*	Desert edible mushroom	Coastal, barren, and desert soils	Edible	Heat tolerance; deep rhizoidal anchorage [36].
*Pleurotus citrinopileatus*	Golden oyster mushroom	Tropical and subtropical regions, grow on decaying wood	Edible	Broad substrate plasticity; rapid colonization [37].
*Pleurotus eryngii*	King oyster mushroom	Native to Mediterranean regions; grows on decaying roots of herbaceous plants	Edible	Root-associated growth; drought tolerance [38].
*Pleurotus ostreatus*	Oyster mushroom	Temperate and tropical forests grow on decaying wood	Edible	Ligninolytic enzyme production; climate adaptability [39].
*Pleurotus pulmonarius*	Phoenix oyster mushroom	Widely distributed, grows on dead hardwoods, tolerates warmer temperatures	Edible	Heat tolerance; wide geographic distribution [40].
*Pleurotus sajor-caju*	Grey oyster mushroom	Tropical and subtropical climates, grows on various lignocellulosic materials	Edible	Substrate versatility; rapid fruiting [41].
*Podaxis pistillaris*	Desert shaggy mane	Arid and semi-arid regions worldwide	Edible	Heat tolerance; xerophytic sporulation [42].
*Russula emetica*	The sickener	Temperate and boreal woodlands	Inedible	Short-lived sporocarps; mycorrhizal dependence [43].
*Schizophyllum commune*	Split gill mushroom	Wide range of habitats, including arid regions, grows on decaying wood	Edible	Extreme desiccation tolerance; rapid rehydration [44].
*Suillus luteus*	Slippery jack	Coniferous forests; pine associations	Edible	Pine-specific mycorrhiza; carbon exchange efficiency [45].
*Termitomyces striatus*	-	Tropical and subtropical regions, associated with termite mounds	Edible	Fungus–termite symbiosis; nutrient recycling [46].
*Termitomyces titanicus*	Termite mushroom	African tropical regions, associated with termite mounds	Edible	Obligate termite association; large sporocarp formation [47].
*Tricholoma matsutake*	Matsutake	Pine forests in temperate zones	Edible	Mycorrhizal specialization; forest soil adaptation [48].
*Tulostoma brumale*	Winter stalkball	Arid and semi-arid regions, sandy soils	Inedible	Stalked puffball morphology; arid survival [49].
*Volvariella volvacea*	Straw mushroom	Tropical and subtropical regions	Edible	Rapid life cycle; thermophilic growth [50].

## Data Availability

No new data were created or analyzed in this study. Data sharing is not applicable to this article.
